# Biodegradable Temperature Sensors with Enhanced Sensitivity Using Bioderived Ionic Liquid with Sodium Ions

**DOI:** 10.1021/acsami.5c04965

**Published:** 2025-07-01

**Authors:** Shunsuke Yamada, Takashi Honda

**Affiliations:** Department of Electrical and Electronic Engineering, School of Engineering, 12924 Kyushu Institute of Technology, 1-1 Sensuicho, Tobata Ward, Kitakyushu, Fukuoka 804-8550, Japan

**Keywords:** ionic liquid, transient electronics, temperature sensor, bioderived materials, biodegradability

## Abstract

Gels promise the development of flexible sensors for electronic and ionic skins owing to their high affinity for human skin, making them ideal for biomedical monitoring. Bioderived ionic liquids and their gels possess favorable physical and electrochemical characteristics, including extremely low vapor pressure, high ionic conductivity, and biodegradability, which suit applications of transient electronics requiring the autonomous decomposition of materials. However, their low temperature coefficient of resistance (TCR) may hinder the use of bioderived ionic liquids in wearable devices, implants, and environmental sensing. To address this issue, we doped sodium (Na) ions into the bioderived ionic liquid choline lactate to enhance its TCR for biodegradable temperature sensors. The Na ions improve the coupling of choline and lactate, resulting in a Na-doped ionic liquid with a high TCR, which is attributed to enhanced intermolecular interactions. The ionic gel, composed of the Na-doped ionic liquid and poly­(vinyl alcohol), demonstrated a high thermal index of 7563 K, an activation energy of 1303 meV, and a TCR of 8.4%/K. The temperature sensor degraded in phosphate-buffered solution over 48 days, leaving the encapsulation layer. This study provides insight into enhancing the sensitivity of temperature sensors with ionic liquids for healthcare, wearable, and environmental applications.

## Graphical abstract

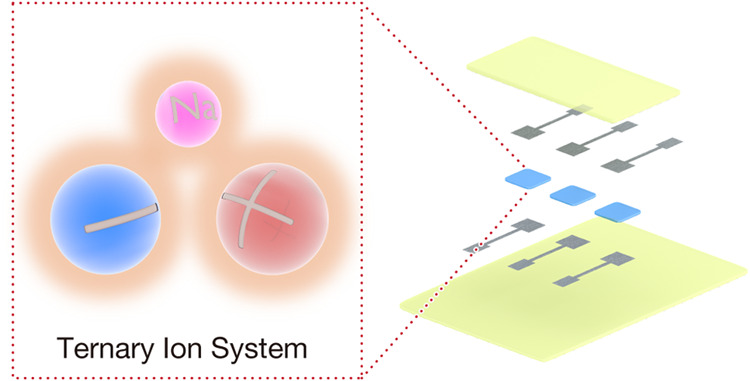

## 1 Introduction

Soft materials with low Young’s moduli provide electronic devices with a high affinity for human skin, allowing artificial skin to imitate the function of human skin, known as electronic skin (e-skin). E-skin has attracted immense attention in electronics, mechanical engineering, and materials science.^1–3^ By adopting organic and inorganic materials, e-skins have developed essential components for electronics, including transistors,^4–6^ sensors,^7–9^ and radio frequency circuits.^10–12^ The successful development of e-skin enables wearables, implants, and soft robots for human–machine interaction that is unachievable with conventional electronics. While e-skins primarily utilize electrons as carriers for device operation, ions serve as alternative carriers for a human–machine interface, leading to the development of ionic skins (i-skins).^13–15^ The i-skins comprise metals, gels, and polymers serving as electronic conductors, ionic conductors, and insulators, respectively. Hydrogels are widely used as electrolytes for sensors,^16–18^ electrochemical transistors,^19–21^ and energy storage devices^22–24^ owing to their excellent mechanical and ionic characteristics, as well as biocompatibility.^25–30^ In addition to hydrogels, an emerging class of electrolytes—ionic liquids (ILs), have been used for i-skins. Comprising pairs of ions, ILs exhibit high ionic conductivity (∼mS/cm),^31–33^ large potential windows,^34^ and extremely low vapor pressure,^35^ all of which are beneficial for i-skins. Most ILs are toxic, which may hinder applications requiring compatibility with human bodies and minimal environmental impact.^36,37^ Therefore, we employed a bioderived IL and its gel electrolytes, named ionic gels (IGs), to develop biodegradable, biocompatible, and flexible i-skins.^38–43^ The hydrogels and IGs exhibit varying ionic conductivities in response to temperature changes,^44^ making them suitable for temperature-sensing applications. However, both hydrogels^45,46^ and IGs^47^ have a low temperature coefficient of resistance (TCR) of less than 5%/K, which may hinder the sensing application of IGs for i-skins.

Herein, we doped sodium (Na) ions into a bioderived IL, choline lactate ([Ch]­[Lac]), to enhance TCR for biodegradable temperature sensors, addressing the issue of low TCR (Figure 1a). ILs can dissolve salts that share the same cations or anions, thereby forming a ternary ion system.^48–51^ The [Ch]­[Lac] is readily biodegradable and a promising candidate for disposable sensors postuse.^38^ Furthermore, lactate forms various salts, such as lithium lactate ([Li]­[Lac]),^52^ sodium lactate ([Na]­[Lac]),^53^ and calcium lactate ([Ca]­[Lac]),^54^ allowing [Ch]­[Lac] to maintain its biodegradability in ternary ion systems ([Ch]­[Lac]­[X], X = Li, Na, or Ca).^39^ While [Ch]­[Lac] remains in a liquid state at room temperature (∼25 °C), the [Na]­[Lac] is solid owing to its high melting point of 437 K (164 °C).^53^ This high melting point indicates strong ionic coupling between Na and lactate, resulting in the ternary system [Ch]­[Lac]­[Na], which exhibits high TCR owing to enhanced intermolecular interactions (Figure 1b). As the mole percent *M*_p_ of Na ions increases, the IL containing 10 mol % Na ions (ILNa10) exhibits the highest thermal index *B* of 7222 K, a thermal activation energy *E*_a_ of 1245 meV, and a TCR of 8.0%/K. We synthesized IGs (IGNa10) by dispersing ILNa in poly­(vinyl alcohol) (PVA), as PVA is widely recognized as a biodegradable polymer and is commonly used as a substrate or gel matrix in transient electronics.^55,56^ The temperature sensor with IGNa10 has a *B* of 1303 meV, an *E*_a_ of 7563 K, and a TCR of 8.4%/K. Encapsulation with the ester bond, cross-linked photo-cross-linked poly­(octamethylene maleate (anhydride) citrate) (EPPOMaC) ensures stable operation of the temperature sensor in ambient air. Degradation tests on the temperature sensor revealed that molybdenum (Mo) foil and IGNa10 dissolved in a 10 mM phosphate-buffered solution (PBS), leaving the EPPOMaC substrate intact.

**Figure 1:**
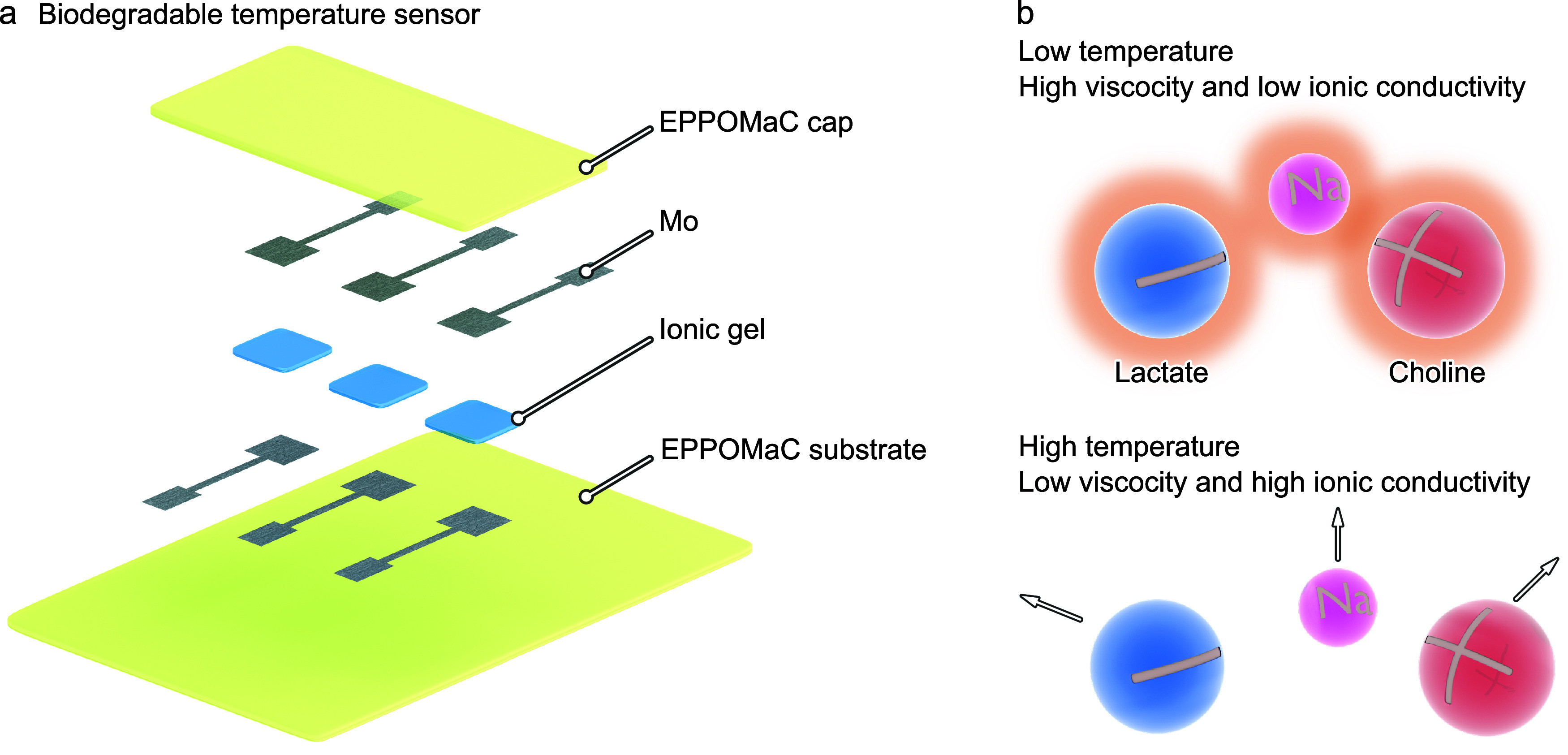
Schematic illustration and sensing mechanism of the temperature sensors. (a) The temperature sensor comprises Mo, IGs, and EPPOMaC, serving as electrodes, sensing materials, and substrates, respectively. The EPPOMaC layers encapsulate the whole structure to protect the IGs from moisture in ambient air. (b) Coupled ions dissociate to either decrease or increase the ionic conductivity of the IGs at low or high temperatures. Na ions enhance the ionic coupling, leading to a high-temperature coefficient of resistance.

## 2 Results and Discussion

### 2.1 Temperature Characteristics of ILs with Sodium Ions

We synthesized IL following the protocol reported by Migliorini et al.,^57^ after which [Na]­[Lac] was dissolved in the IL. The Na-ion *M*_p_ is defined as follows

1 $$M_{p}=\frac{\left[\mathrm{Na}\right]\left[\mathrm{Lac}\right]}{\left[\mathrm{Ch}\right]\left[\mathrm{Lac}\right]+\left[\mathrm{Na}\right]\left[\mathrm{Lac}\right]}\times 100\;\left(\mathrm{mol}\;\%\right)$$

where [Na]­[Lac] and [Ch]­[Lac] are the moles of sodium lactate and IL, respectively. We prepared ILs containing Na ions with *M*_p_s of 5 mol % (ILNa5), 10 mol % (ILNa10), and 20 mol % (ILNa20) to investigate the influence of Na-ion *M*_p_ on the IL characteristics, following the methodology reported in our previous studies. For reference, the pure IL is referred to as a pristine IL (PIL) hereafter. We injected the ILs into glass containers equipped with a pair of electrodes, with a glass spacer maintaining an electrode gap.^39^ The glass container was placed on a thermoelectric heater using two Pt100 sensors to control and monitor the temperatures of the ILs (Figure 2a). We performed electrochemical impedance spectroscopy (EIS) measurements on the PIL, ILNa5, ILNa10, and ILNa20 at temperatures ranging from 25 to 80 °C. The EIS curves at 25 °C show high impedance |*Z*| in low-frequency regimes and low impedance in high-frequency regimes, while phase shifts θs are low in low-frequency regimes and high in high-frequency regimes (Figure 2b–e). As the temperature increased, the impedance of the ILs in the high-frequency regime decreased, while the phase shift increased with frequency ranging from 100 Hz to 1 kHz. These changes in the EIS curves result from ion dissociation and a reduction in the viscosity of the ILs.

**Figure 2:**
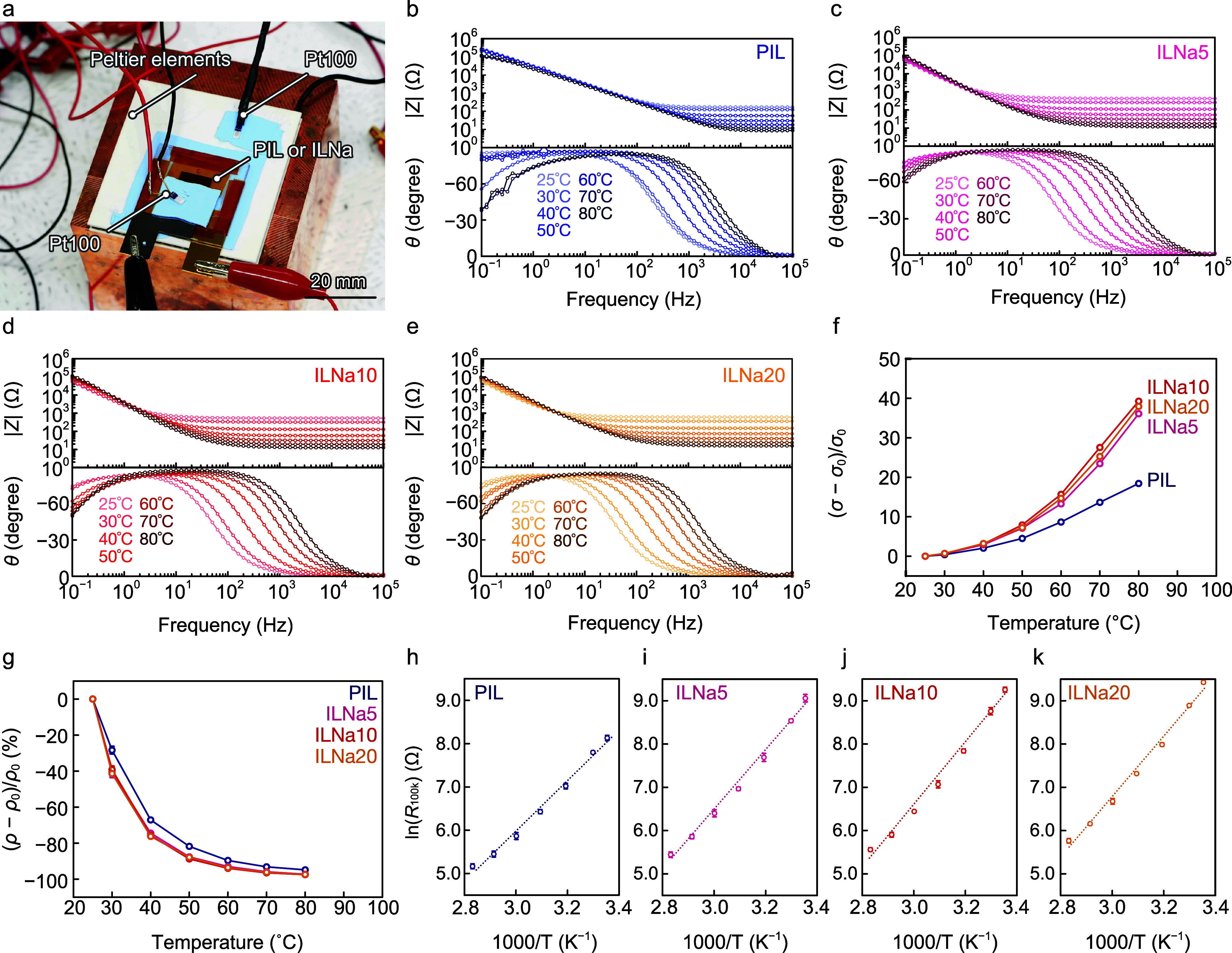
Investigation of the temperature dependence of IG characteristics. (a) Experimental setup for electrochemical impedance spectroscopy (EIS) measurements with temperature control. EIS spectra for (b) PIL, (c) ILNa5, (d) ILNa10, and (e) ILNa20 at temperatures ranging from 25 to 80 °C. Changes in (f) ionic conductivity and (g) resistivity of the ILs were observed at 100 kHz. Plots of ln­(*R*_100k_) versus 1000/*T* for (h) PIL, (i) ILNa5, (j) ILNa10, and (k) ILNa20.

The ionic conductivity σ of ILs at 100 kHz is expressed as follows

2 $$\sigma =\frac{t}{AR_{100k}}$$

where *t*, *A*, and *R*_100k_ present the gap between the electrodes, the contact area between the IL and the electrodes, and the IL resistance at 100 kHz, respectively.^58^ We summarized the changes in σ with temperature (Δσ) and defined as follows

3 $$\Delta \sigma =\frac{\sigma -\sigma_{0}}{\sigma_{0}}$$

where σ_0_ is the σ at 25 °C. While the Δσ of PIL is 18.4 for temperatures ranging from 25 to 80 °C, the Δσ values for ILNa5, ILNa10, and ILNa20 are 35.0, 40.8, and 37.0, respectively (Figure 2f). Na-ion doping enhances Δσ, maximizing at an *M*_p_ of 10 mol %. IL resistivity ρ is defined as follows

4 $$\rho =\frac{1}{\sigma }$$

The Δσ can be rewritten as a change in resistivity, Δρ, defined as follows

5 $$\Delta \rho =\frac{\rho -\rho_{0}}{\rho_{0}}$$

where ρ_0_ denotes the ρ at 25 °C. The ILNa5, ILNa10, and ILNa20 exhibit larger Δρ values compared to PIL for temperatures ranging from 25 to 80 °C (Figure 2g). The *R*_100k_ for the ILs at temperature *T* is expressed as follows

6 $$R_{100k}=R_{0}\;\exp\left(\frac{E_{a}}{2\mathrm{kT}}\right)$$

where *R*_0_, *E*_a_, and *k* represent the resistance at infinite temperature, the thermal activation energy, and the Boltzmann constant, respectively.^59,60^ The *E*_a_ can be rewritten using thermal index *B* as follows

7 $$E_{a}=2\mathrm{kB}$$

Taking the natural logarithm of both sides of eq 6 yields

8 $$\ln\left(R_{100k}\right)=\ln\left(R_{0}\right)+\frac{E_{a}}{2\mathrm{kT}}=\ln\left(R_{0}\right)+\frac{B}{T}$$

Equation 8 indicates a linear correlation between 1/*T* to ln­(*R*_100k_). We plotted ln­(*R*_100k_) versus 1000/*T* for the ILs and applied the least-squares method to fit the data with dotted lines (Figure 2h–k). The fitting curves align well with the experimental results for PIL, ILNa5, ILNa10, and ILNa20. Based on the fitting curves, the *B* values for PIL, ILNa5, ILNa10, and ILNa20 were calculated to be 5845, 6914, 7200, and 7024 K, respectively. The corresponding *E*_a_ values for PIL, ILNa5, ILNa10, and ILNa20 were determined to be 1007, 1192, 1241, and 1211 meV, respectively, using eq 7. To evaluate TCR, we defined TCR as follows

9 $$\mathrm{TCR}=\frac{dR_{100k}}{dT}\times \frac{1}{R_{100k}}$$

Substituting *R*_100k_ from eq 6 yields

10 $$\mathrm{TCR}=-\frac{B}{T^{2}}$$

Using eq 10, the TCR values for PIL, ILNa5, ILNa10, and ILNa20 at 300 K were calculated to be 6.49, 7.64, 8.00, and 7.80%/K, respectively. The *R*_100k_ values of PIL, ILNa5, ILNa10, and ILNa20 were 170, 429, 523, and 620 Ω at 25 °C, respectively, while the corresponding values at 80 °C were 8.76, 11.6, 13.0, and 15.9 Ω. The impedance *R*_100k_ at 25 °C reached 2.52-, 3.07-, and 3.65-fold higher values for ILNa5, ILNa10, and ILNa20, respectively, relative to PIL (170 Ω). At 80 °C, the *R*_100k_ values for ILNa5, ILNa10, and ILNa20 were approximately 1.32-, 1.48-, and 1.82-fold higher, respectively, than that of PIL (8.67 Ω). This suggests that although impedance increases with Na content, the greatest temperature-dependent impedance change—reflecting thermal sensitivity, occurs at 10 mol % Na. At higher Na concentrations (e.g., ILNa20), excess Na^+^ ions appear to increase the impedance at 80  °C, likely due to ionic aggregation^61^ or reduced ion mobility,^62^ which limits the temperature-responsive behavior. We summarize the *B*, *E*_a_, and TCR values of the ILs in Table 1. The 10 mol % Na-ion concentration yielded the maximum *B*, *E*_a_, and |TCR| values, with each increasing by 23.2% relative to those of the PIL.

**Table 1:** Summary of the Thermal Index, Thermal Activation Energy, and Temperature Coefficient of Resistance for PIL, ILNa5, ILNa10, and ILNa20

No.	ionic liquid	thermal index *B* (K)	thermal activation energy *E*_a_ (meV)	temperature coefficient of resistance |TCR| (%/K)
1	PIL	5845	1007	6.49
2	ILNa5	6914	1192	7.64
3	ILNa10	7200	1241	8.00
4	ILNa20	7024	1211	7.80

### 2.2 Characterization of IGs

We developed IGNa10 by dispersing ILNa10 in PVA (Figure 3a) and investigated its optical, thermal, and mechanical characteristics for temperature sensor applications. The developed IGNa10 is transparent (Figure 3b), with ultraviolet–visible (UV–vis) spectrophotometry confirming a transmittance of over 86% in the visible spectrum (400–800 nm, Figure 3c). A differential scanning calorimetry (DSC) curve indicates that IGNa10 has a glass transition temperature of 203.65 K (−69.5 °C). No endothermic or exothermic peaks were observed from 243.15 K (−30 °C) to 353.15 K (80 °C) in the DSC curve, demonstrating the thermal stability of the sensor within its operating temperature range of 30–80 °C (Figure 3d). Tensile stress tests revealed that IGNa10 exhibits high stretchability (Figure 3e), with a fracture strain, stress, and Young’s modulus of 268%, 629, and 228 kPa (Figure 3f), based on triplicate averages. Compared with Young’s modulus, fracture stress and strain of sensing materials in previous works (Table S1, Supporting Information), these mechanical properties are suitable for flexible and stretchable sensors employing IGs. Investigation of the mechanical behavior of IGNa10 at different temperatures remains an important future task to fully establish its suitability for temperature-sensing applications.

**Figure 3:**
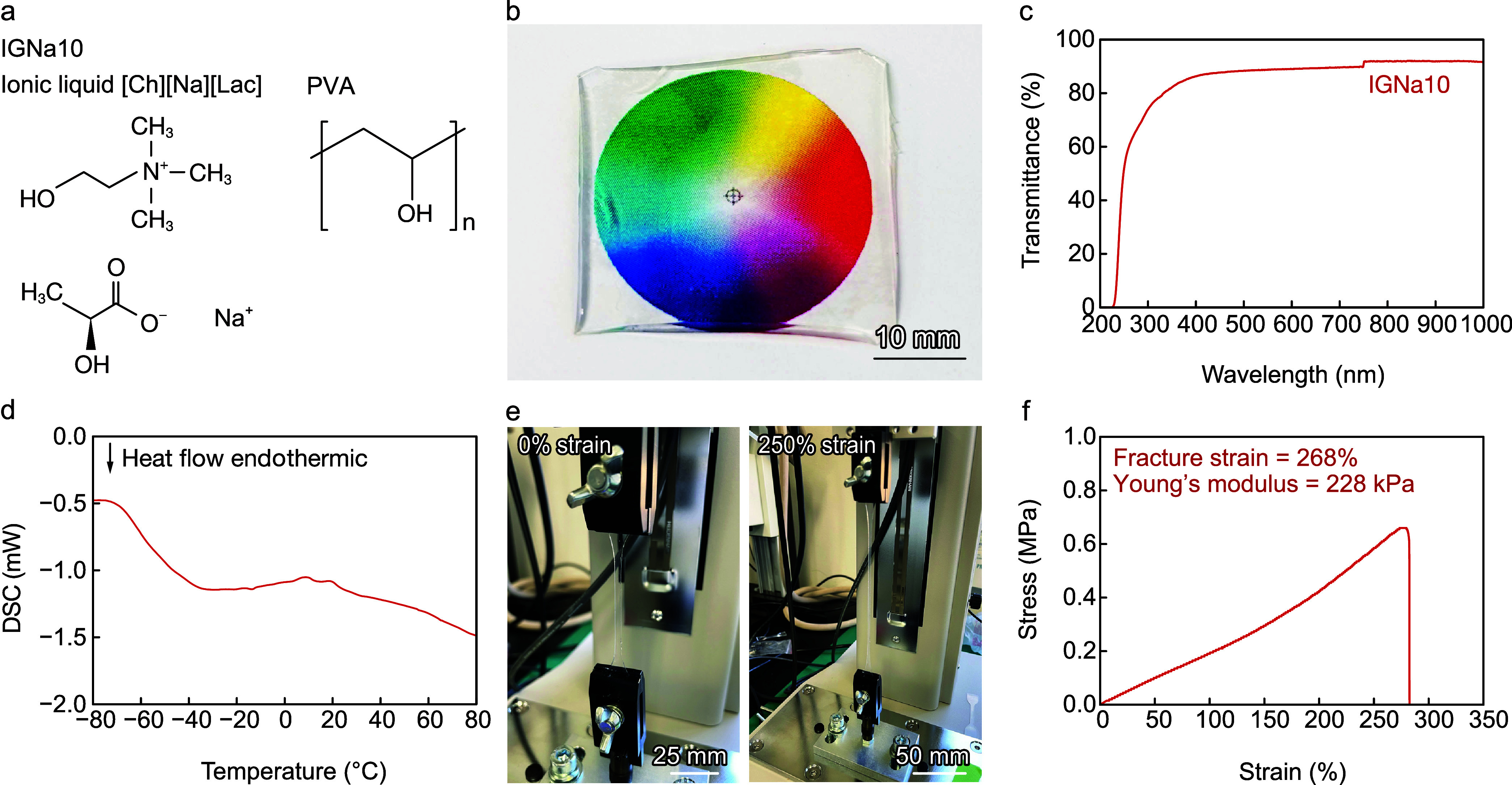
Characterization of IGNa10. (a) Chemical formulas of ILNa10 and PVA. (b) Photograph of the IGNa10. (c) Optical transparency of IGNa10. (d) DSC profile of IGNa10. (e) Photographs of IGNa10 under 0 and 250% strain. (f) Tensile stress curve of IGNa10, with fracture strain and Young’s modulus at 268% and 228 kPa, respectively.

### 2.3 Temperature Sensor with IGNa10

We developed temperature sensors using molybdenum (Mo) foil as electrodes, IGNa10s as electrolytes, and EPPOMaC as substrates (Figure 4a). The PIL and ILNa used in this study are anhydrous and therefore do not induce electrochemical reactions with the Mo electrodes during device operation, enabling stable sensor performance. EIS measurements of the IGNa10 were conducted over a temperature range of 25–80 °C. While |*Z*| exhibited high values at 0.1 Hz and low values at 100 kHz, θ showed the opposite trend, with low values at 0.1 Hz and high values at 100 kHz. A similar trend was observed in ILNa10 (Figure 4b), suggesting that IGNa10 effectively functions as an ionic conductor. As the IGNa10 temperature increased, its |*Z*| in the high-frequency range (>1 kHz) decreased, attributed to the dispersion of ILNa10 within the PVA matrix, confirming the suitability of IGNa10 as a temperature-sensing material. While the |*Z*| of ILNa10 in the low-frequency range (0.1–10 Hz) displayed minimal temperature dependence, IGNa10 demonstrated higher impedance at 25–50 °C compared to 60–80 °C. The dispersion of ILs in the polymer resulted in its reduced ionic conductivity, leading to increased impedance in IGNa10. At 80 °C IGNa10 Δσ reached 49, exceeding the ILNa10 Δσ of 41 (Figure 4c). Given the intrinsic thermal stability and consistent performance of the ionic-liquid-based material, the variation in repeated measurements is anticipated to be negligibly small. Accordingly, Figure 4c–e present representative data without error bars. Both materials exhibited similar Δρ curves (Figure 4d). We plotted ln­(*R*_100k_) versus 1000/*T* for the temperature sensor and drew dotted lines, applying the least-square method to the data (Figure 4e). The experimental results matched the fitted curve, yielding a *B* of 7563 K, an *E*_a_ of 1303 meV, and a TCR of 8.4%/K at 300 K, calculated using eqs 6, 7, and 9. The TCR value in this study is notably higher than previously reported values for sensors using ILs and comparable to those of a deep eutectic gel, indicating the high sensitivity of the IGNa10-based temperature sensor (Table S2 and Figure S1, Supporting Information). We evaluated the response of the temperature sensor. The *R*_100k_ of IGNa10 was 30 kΩ at room temperature (∼25 °C). When the IGNa10 contacted the hot plate surface at 40 °C, its impedance reduced from 31 to 9.8 kΩ within 1 s, continuing to decrease as the temperature of the sensor stabilized (Figure 4f). This result indicates a rapid response to temperature variation. This result indicates a rapid response to temperature variation. The temperature sensor exhibited a negligibly small relative change in conductivity over the applied pressure range up to 50 kPa at 25 °C, indicating that it operates accurately and is decoupled from pressure interference (Figure S2, Supporting Information).

**Figure 4:**
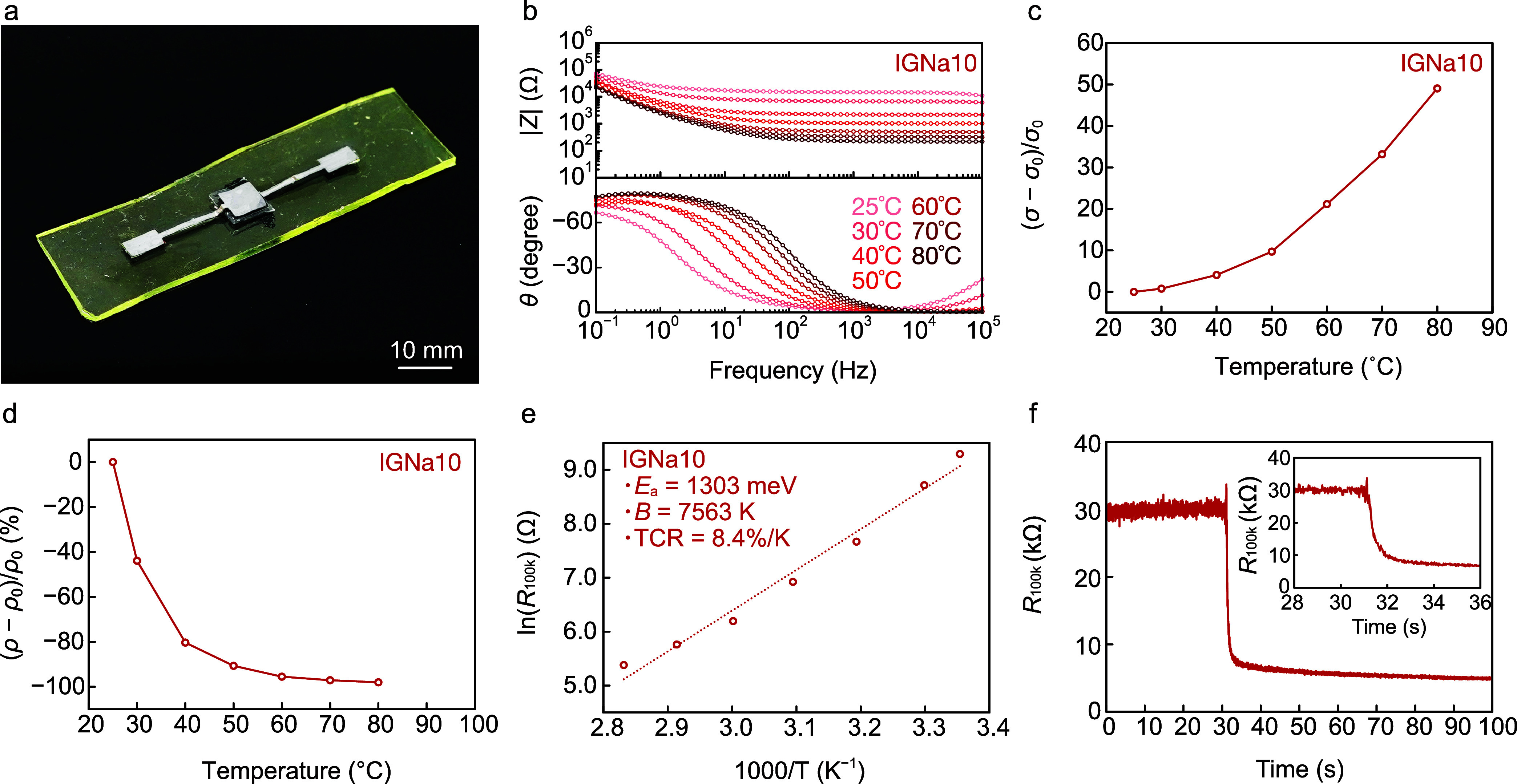
Characterization of the temperature sensor with an IGNa10. (a) Photograph of the temperature sensor. (b) EIS spectra of the temperature sensor across temperatures ranging from 25 to 80 °C. Change in (c) ionic conductivity and (d) resistivity of the temperature sensor at 100 kHz. (e) Plots of ln­(*R*_100k_) versus 1000/*T* for the temperature sensor. (f) Response of the temperature sensor, with the inset showing a magnified view at 28–36 s.

### 2.4 Temperature Sensors for Multiple Point Sensing in Ambient Air

Although the ILs and IGNa10 show high sensitivity, their hygroscopic nature makes them prone to changes in the ionic conductivity in ambient air. To mitigate moisture absorption, we encapsulated the three IGNa10s using an EPPOMaC sheet (Figure 5a). The precursor of EPPOMaC served as an adhesive, with UV and heat curing securely bonding the two EPPOMaC sheets (Figure 5b). The encapsulated temperature sensors were remained flexible, allowing them to conform well to curved surfaces (Figure 5c). The Δσ values of the left, middle, and right sensors at room temperature were −0.12, −0.13, and −0.14, respectively. Upon finger touch, the Δσ values for the left and right sensors increased to 0.38 and 0.41, respectively, while the middle sensor showed no change, demonstrating multipoint temperature sensing (Figure 5d,e). We evaluated the transient temperature behavior of IGNa10 during finger contact on the right sensor by using eq 6. The temperature of the sensor gradually rose from 23.2 to 29.0 °C within 60 s of contact (Figure 5f). Compared to the unencapsulated sensor, the encapsulated version showed a slower temperature response owing to the low thermal conductivity of the EPPOMaC layer. In this work, the thickness of the EPPOMaC capping layer was fixed at approximately 1 mm. Reducing the thickness of the EPPOMaC encapsulation and substrate layers is expected to lower thermal resistance and enhance the response time of the sensor, which will be the focus of future optimization efforts. After removal of the finger, the temperature of the sensor slowly returned to 22 °C over 900 s (Figure 5g). We measured the ionic conductivity of the sensor to evaluate its thermal stability by cycling the temperature between 25 and 80 °C for five cycles. The ionic conductivity remained stable throughout the temperature range, showing no significant change. This result indicates that neither the IGNa10 nor the Mo electrode underwent thermal decomposition or oxidation that would degrade the sensor’s performance (Figure S3, Supporting Information). This sensor demonstrated repeatable and stable operation under ambient conditions, confirming that EPPOMaC encapsulation effectively mitigates moisture-induced degradation. Although EPPOMaC is a relatively hydrophilic material and allows for gradual moisture ingress over time, it still provides partial protection. A sensor without EPPOMaC encapsulation, stored at room temperature (∼25 °C) for 5 days, exhibited noticeable changes in the EIS spectra due to moisture absorption by the IGNa10. In contrast, the sensor with EPPOMaC encapsulation showed a significantly smaller shift in the EIS curves, indicating that the encapsulation layer suppressed moisture uptake and preserved the ionic gel’s properties (Figure S4, Supporting Information). However, the sensing performance may be degraded during long-term use. To address this issue, hydrophobic electrolytes represent a promising alternative owing to their intrinsic ability to repel ambient moisture. Zhang et al. reported strain sensors for underwater applications using hydrophobic eutectogels produced from a deep eutectic solvent (DES) and monomers. The eutectogels exhibited a contact angle greater than 110°, demonstrating excellent water resistance with minimal changes in ionic conductivity upon immersion.^63^ Deep eutectic solvents or ionic liquids composed of hydrophobic components may offer enhanced stability for temperature sensor operation.

**Figure 5:**
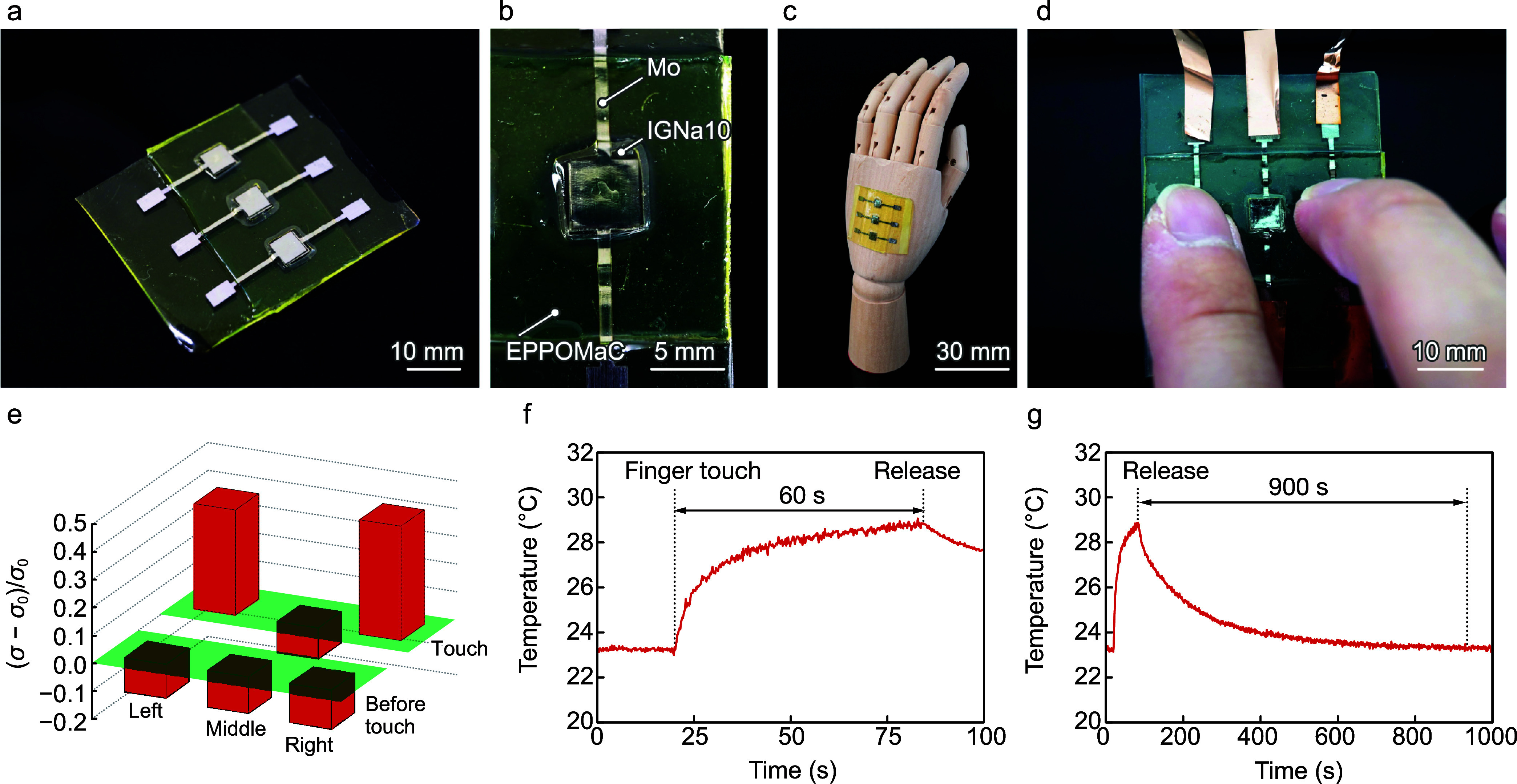
Array of encapsulated temperature sensors for multiple sensing. Photographs of (a) the three temperature sensors and (b) a magnified image of a temperature sensor encapsulated with an EPPOMaC layer. (c) Conformability of the temperature sensor to a curved surface. (d) Multipoint temperature sensing in response to finger touch. (e) Corresponding changes in σ of the sensors and their transient behavior on (f) short and (g) long time scales.

### 2.5 Sensor Degradation in PBS Solution

We previously assessed the biodegradation of ILs and those containing Na ions via a modified protocol of the Organization for Economic Co-operation and Development (OECD) 301C MITI test (I), which revealed their ready biodegradability.^38,39^ Consequently, we investigated the degradation of the Mo electrode and the unencapsulated temperature sensor by immersing them in 100 mL of 10 mM PBS solution at 37 °C. The Mo electrode initially showed a glossy silver-gray appearance that gradually turned dark gray on day 63 (Figure 6a). Ultimately, the Mo foil finally ruptured, leaving behind tiny fragments of Mo. The change in the mass of the Mo foil (*M*_c_) during degradation is expressed as the ratio of its mass *M* to its initial mass *M*_0_, defined by using the following equation

11 $$M_{c}=\frac{M-M_{0}}{M_{0}}\times 100\;\left(\%\right)$$

**Figure 6:**
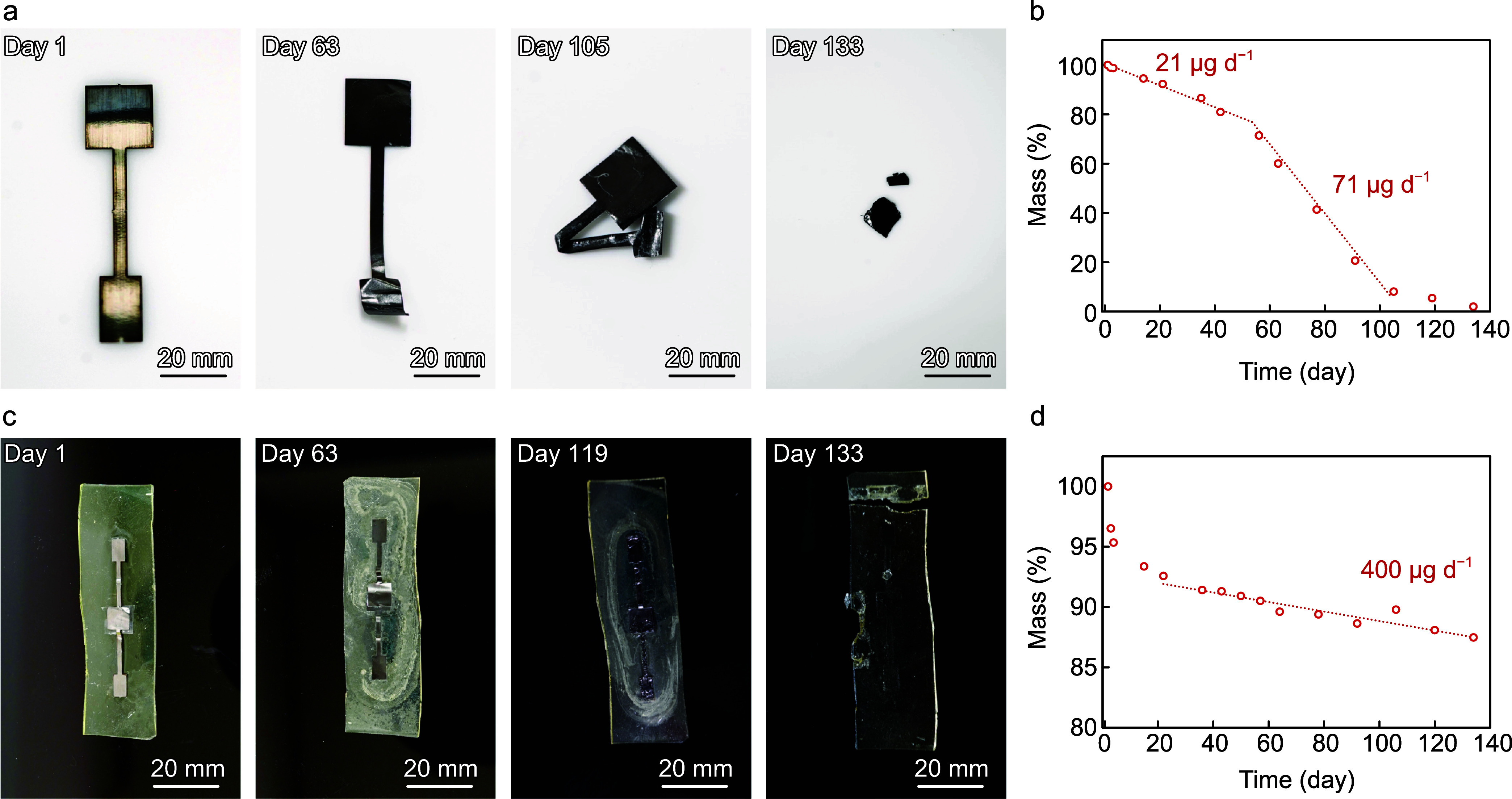
Degradation test on Mo electrodes and a temperature sensor. (a) Time-lapse photographs of a Mo electrode soaked in PBS solution at 37 °C and (b) the corresponding mass change over time. (c) Time-lapse photographs of a whole temperature sensor without a capping layer degraded in PBS solution at 37 °C and (d) corresponding mass change over time.

The Mo dissolution kinetics remained relatively slow until day 49, then accelerated from day 56 to day 133 (Figure 6b), with the slow and fast dissolution rates measured at 0.43%/day (21 μg/day) and 1.4%/day (71 μg/day), respectively. In a previous study, we reported that Mo electrodes coated with MoO_3_ showed a high dissolution rate of 320 μg/day.^38^ The results in this study suggest that Mo dissolution involves two dissolution kinetics: hydrolysis of Mo (slow) and oxidized Mo (fast), described as follows

Mo hydrolysis^64,65^

12 $$\mathrm{Mo}+4H_{2}O\to \mathrm{Mo}O_{4}^{2-}+8H^{+}+6e^{-}$$

Hydrolysis of oxidized Mo^66^

13 $$\mathrm{Mo}O_{2}+2H_{2}O\to \mathrm{Mo}O_{4}^{2-}+4H^{+}+2e^{-}$$

or

14 $$\mathrm{Mo}O_{3}+2{\mathrm{OH}}^{-}\to \mathrm{HMo}O_{4}^{-}+H_{2}O+e^{-}$$

While some Mo dissolved in the PBS solution during the early stage, another part began to oxidize to MoO_2_ or MoO_3_ via the following reactions.^67^

Mo oxidation

15 $$\mathrm{Mo}+2H_{2}O\to \mathrm{Mo}O_{2}+4H^{+}+4e^{-}$$

and

16 $$\mathrm{Mo}+3H_{2}O\to \mathrm{Mo}O_{3}+6H^{+}+6e^{-}$$

By day 56, the oxidation reactions had formed an oxide layer on the Mo surface, altering its color and accelerating the dissolution rate of the Mo electrode. The Mo dissolution rates of 21 and 71 μg/day are favorable for implants, as they are lower than the average daily Mo intake for humans (300 μg).^68^ In the dissolution test of the complete temperature sensor without a capping layer, the EPPOMaC maintained stable chemical bonding and ester cross-linking, remaining intact, whereas the Mo electrodes and IGNa10 dissolved into the PBS at a dissolution speed of 400 μg/day (Figure 6c,d). Although EPPOMaC did not fully degrade during the test, its components—1,8-octanediol, citric acid, and maleic anhydride—are biodegradable, with previous studies reporting its in vivo degradation behavior.^8,69^

## 3 Conclusions

Biodegradable temperature sensors were developed by adopting bioderived ILs with Na ions. The Na ions reduced the ionic conductivity of the ILs at 30 °C, while ion dissociation enhanced the temperature dependence of the ionic conductivity. The ILNa10 showed *B*, *E*_a_, and TCR of 7222 K, 1241 meV, and 8.0%/K, respectively, which are higher than and comparable to previous studies. The IGNa10, composed of ILNa10 and PVA, showed *B*, *E*_a_, and TCR of 7563 K, 1303 meV, and 8.4%/K, respectively. The array of three temperature sensors achieved multipoint temperature sensing, and EPPOMaC encapsulation allowed the sensor to function in ambient air without altering the ionic conductivity. The temperature sensor dissolved in PBS solution within 133 days, leaving behind the EPPOMaC substrate. This study provides insight into enhancing the sensitivity of temperature sensors by using ILs and affords disposable sensors for applications in healthcare, wearables, and environmental sensing.

## 4 Experimental Section

### 4.1 Synthesis of Ionic Liquid and Sodium Ion Doping

Choline bicarbonate (product ID: C7519, Merck) was slowly poured into lactic acid (product ID: 27715, Merck) in a molar ratio of 1:1 and stirred for 24 h with a magnetic stirrer. A rotary evaporator removed water from the solution heated at 50 °C and ∼5 kPa until bubbling stopped. The solution was then vacuum-annealed at 50 °C and ∼10 Pa to remove residual moisture. Sodium l-lactate (product number: 71718-10G, Sigma-Aldrich) was dissolved in a small amount of deionized water (DIW), and the IL and sodium l-lactate solutions were mixed, resulting in mole percents of Na ions at 5, 10, and 20 mol % relative to the ILs. The mixture was stirred for 24 h with a magnetic stirrer followed by vacuum-annealing at 50 °C and 5 Pa for another 24 h to remove water.

### 4.2 Preparation and Characterization of the Ionic Gel

We mixed ILNa10, PVA (product ID: 341584-500G; *M*_w_ 89,000–98,000; >99% hydrolyzed), and DIW in a weight ratio of 7:3:18. The PVA was purchased from Sigma-Aldrich. The mixture was added into a glass bottle and heated at 110 °C to obtain a clear solution. The solution was blade-coated on a glass plate with a blade height of 1 mm and allowed to gelatinize under ambient conditions (∼24 h). The IGNa10 was then cut and vacuum-annealed at 50 °C and 5 Pa for 24 h to remove water before use, yielding an IGNa10 film with a thickness of approximately 100 μm. A UV–vis spectrophotometer (V700, JASCO) measured the transparency of the IGNa10. The thermal profile was obtained with a differential scanning calorimeter (DSC8230/TG8 120, Rigaku).

### 4.3 Device Fabrication and Characterization

Mo foil with a thickness of 10 μm was cut using a laser processing machine (ProtoLaser U4, LPKF Laser & Electronics KK), followed by soaking in a 29% ammonia solution to remove oxide layers formed during laser cutting. The Mo foils and IGNa10s were transferred onto EPPOMaC substrates to form a Mo–IGNa10–Mo configuration. We coated the precursor of EPPOMaC onto a single side of the EPPOMaC and laminated it onto the EPPOMaC substrate containing the Mo foils and IGNa10s. The EPPOMaC was heated at 100 °C for 6 h to complete polymerization, yielding encapsulated temperature sensors. The temperature sensors were characterized using an EIS module (FRA32M, Autolab), while an impedance analyzer (IM3590, HIOKI) measured their response.

### 4.4 Degradation Test

A 10 mM PBS solution was prepared by dissolving salts (NaCl (8.0 g), KCl (0.2 g), Na_2_HPO_4_·12H_2_O (2.9 g), and KH_2_PO_4_ (2.9 g)) in DIW (1 L). The 10 μm-thick Mo electrode and temperature sensor were immersed in 100 mL of PBS, which was stored in an oven at 37 °C. The solutions were refreshed daily.

## Supplementary Material



## Data Availability

A table and graph of sensor comparison from recent literature are available within the article and the Supporting Information.

## Notes

Comparison of sensing materials, thermal index, thermal activation energy, and TCR from recent studies on temperature sensors (PDF)
