# Supplementary material for: Biodegradable Temperature Sensors with Enhanced Sensitivity Using Bioderived Ionic Liquid with Sodium Ions
Source: ACS Appl Mater Interfaces. 2025 Jul 1;17(28):40845–54. doi: 10.1021/acsami.5c04965 (PMC12278216; doi:10.1021/acsami.5c04965)
Supplement: Supplementary file 1 [file am5c04965_si_001.pdf]

Supporting Information

Biodegradable Temperature Sensors with Enhanced Sensitivity Using Bioderived Ionic Liquid with Sodium Ions

Shunsuke Yamada [a]* and Takashi Honda [a]

1-1 Sensuicho, Tobata, Kitakyushu, Fukuoka 804-8550, Japan

Department of Electrical and Electronic Engineering, Kyushu Institute of Technology

[a] Department of Electrical and Electronic Engineering, School of Engineering, Kyushu Institute of Technology, 1-1 Sensuicho, Tobata Ward, Kitakyushu City, Fukuoka 804-8550, Japan

* E-mail: yamada@ele.kyutech.ac.jp

Table S1. Comparison of mechanical properties of sensing materials and biodegradability from recent studies on temperature sensors.

Sensing materials	Young's modulus	Fracture stress	Fracture strain (%)	Biodegra-dability	Year	Ref.
Ionic gel [Ch][Lac][Na]	228 kPa	629	268	Yes	2024	This work
Hydrogel (NaCl)	5–35 kPa	>10kPa	> 50	No	2023	1
Deep eutectic gels	25.03±2.89–181.55±8.53 MPa	3-13 Mpa	275-320	No	2021	2
Hydrogels (cellulose nanofibrils, NaCl, tannic acid, and glycerol−water solvent PAAm)	23kPa	86 kPa	1430	No	2021	3
Ionic gel [EMIM][NTf_2] and thermal-plastic polyurethane	–	1.2-6.6 MPa	560-960	No	2022	4
$Ti_3C_2T_x$-MXene hydrogel	0.32–0.84 MPa	1.4–2.28 MPa	225–400	No	2019	5
Nickel oxide	–	–	–	No	2023	6
Nickel-cupper composite	–	–	–	No	2018	7
Polyimine-silver nanoparticle composite	–	–	–	No	2023	8
Gold nanoparticles	–	–	–	No	2021	9
Carboxylic styrene butadiene-CNT composite	0.43–1.19MPa	7.07–12.58 MPa	191.95–217.55	No	2020	10
Elastic polyurethane-Graphene	–	57.8±0.7–70.4±0.5 MPa	455±3 – 528±5	No	2020	11
Porous graphene	–	–	–	No	2024	12

Table S2. Comparison of sensing materials, sensing area, response time, thermal index, thermal activation energy, temperature coefficient of resistance, sensing resolution, and aging test from recent studies on temperature sensors.

Sensing materials	Sensing area	Respone time	Thermal index *B* (K)	Thermal activation energy E_a (meV)	TCR (% K^{-1})	Sensing resolution	Aging test	Year	Ref.
Ionic gel [Ch][Lac][Na]	5×5 mm	< 1	1303	7563	8.4	–	–	2024	This work
Hydrogel (NaCl)	–	86.4s (change of 2 °C)	–	–	2.9	0.1°C	7 d in vivo	2023	1
Deep eutectic gels	–	–	1306.52 ± 46.47	7581.05 ± 269.64	8.42 ± 0.30	1°C	–	2021	2
Hydrogels (cellulose nanofibrils, NaCl, tannic acid, and glycerol−water solvent PAAm)	40 × 10 × 1.5 mm	–	–	–	−1.49	–	10 d	2021	3
Ionic gel [EMIM][NTf_2] and thermal-plastic polyurethane	–	–	–	–	−0.012, −0.005, or −0.003	0.1°C	–	2022	4
$Ti_3C_2T_x$-MXene hydrogel	–	–	–	4428	−5.27, −1.11	0.1°C	–	2019	5
Nickel oxide	50 μm wide NiO channel	<50 ms	–	7350	−9.2	–	–	2023	6
Nickel-cupper composite	Total area: 168 mm^2 (length: 156 mm, width: 200 μm)	–	–	–	0.104-0.235	–	–	2018	7
Polyimine-silver nanoparticle composite	30×1.36 × 0.12 mm	–	–	–	0.17	–	–	2023	8
Gold nanoparticles	–	~5 and 10 s (heating and cooling steps)	–	11328	−12.6	0.2°C	–	2021	9
Carboxylic styrene butadiene-CNT composite	–	<10 s	–	5140	-1.636	–	–	2020	10
Elastic polyurethane-Graphene	–	–	–	–	−0.815	–	–	2020	11
Porous graphene	–	<25 s	–	–	−0.06	0.051°C	–	2024	12

Reference

1. Li, Y.; Li, D.; Wang, J.; Ye, T.; Li, Q.; Li, L.; Gao, R.; Wang, Y.; Ren, J.; Li, F., A Temperature-Sensing Hydrogel Coating on The Medical Catheter. *Advanced Functional Materials* **2024,** *34* (10), 2310260.
2. Yao, P.; Bao, Q.; Yao, Y.; Xiao, M.; Xu, Z.; Yang, J.; Liu, W., Environmentally stable, robust, adhesive, and conductive supramolecular deep eutectic gels as ultrasensitive flexible temperature sensor. *Advanced Materials* **2023,** *35* (21), 2300114.
3. Wei, Y.; Xiang, L.; Zhu, P.; Qian, Y.; Zhao, B.; Chen, G., Multifunctional organohydrogel-based ionic skin for capacitance and temperature sensing toward intelligent skin-like devices. *Chem Mater* **2021,** *33* (22), 8623-8634.
4. Jiang, N.; Chang, X.; Hu, D.; Chen, L.; Wang, Y.; Chen, J.; Zhu, Y., Flexible, transparent, and antibacterial ionogels toward highly sensitive strain and temperature sensors. *Chemical Engineering Journal* **2021,** *424*, 130418.
5. Liu, H.; Du, C.; Liao, L.; Zhang, H.; Zhou, H.; Zhou, W.; Ren, T.; Sun, Z.; Lu, Y.; Nie, Z., Approaching intrinsic dynamics of MXenes hybrid hydrogel for 3D printed multimodal intelligent devices with ultrahigh superelasticity and temperature sensitivity. *Nat Commun* **2022,** *13* (1), 3420.
6. Shin, J.; Jeong, B.; Kim, J.; Nam, V. B.; Yoon, Y.; Jung, J.; Hong, S.; Lee, H.; Eom, H.; Yeo, J., Sensitive wearable temperature sensor with seamless monolithic integration. *Advanced Materials* **2020,** *32* (2), 1905527.
7. Tursunniyaz, M.; Agarwal, V.; Meredith, A.; Andrews, J., Hybrid nanomaterial inks for printed resistive temperature sensors with tunable properties to maximize sensitivity. *Nanoscale* **2023,** *15* (1), 162-170.
8. Zou, Z.; Zhu, C.; Li, Y.; Lei, X.; Zhang, W.; Xiao, J., Rehealable, fully recyclable, and malleable electronic skin enabled by dynamic covalent thermoset nanocomposite. *Science advances* **2018,** *4* (2), eaaq0508.
9. Zhao, X.; Guo, J.; Wang, J.; Yan, Y., A Metal Nanoparticle Thermistor with the Beta Value of 10 000 K. *Small* **2023,** *19* (5), 2205136.
10. Lin, M.; Zheng, Z.; Yang, L.; Luo, M.; Fu, L.; Lin, B.; Xu, C., A high-performance, sensitive, wearable multifunctional sensor based on rubber/CNT for human motion and skin temperature detection. *Advanced Materials* **2022,** *34* (1), 2107309.
11. Hu, X.; Tian, M.; Xu, T.; Sun, X.; Sun, B.; Sun, C.; Liu, X.; Zhang, X.; Qu, L., Multiscale disordered porous fibers for self-sensing and self-cooling integrated smart sportswear. *ACS nano* **2019,** *14* (1), 559-567.
12. Yang, Y.; Song, Y.; Bo, X.; Min, J.; Pak, O. S.; Zhu, L.; Wang, M.; Tu, J.; Kogan, A.; Zhang, H., A laser-engraved wearable sensor for sensitive detection of uric acid and tyrosine in sweat. *Nature biotechnology* **2020,** *38* (2), 217-224.

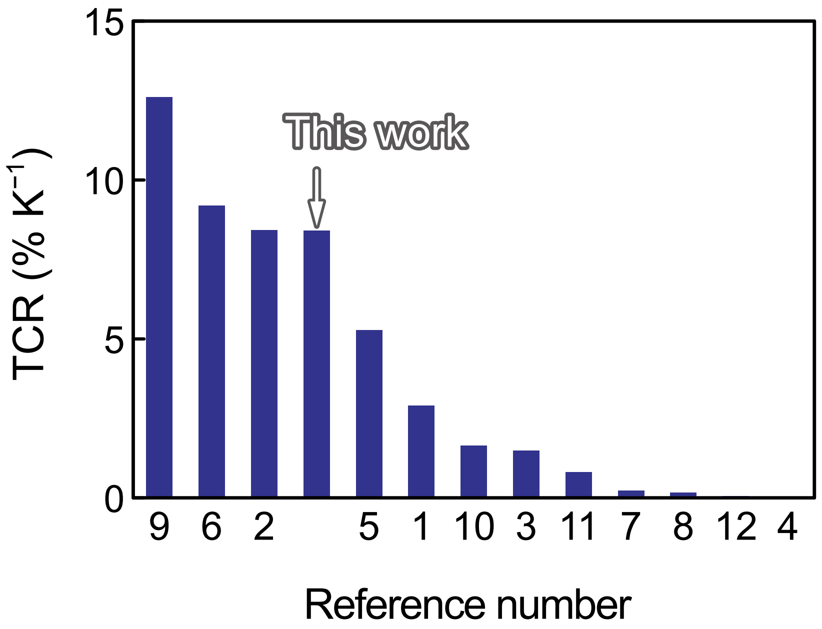

Figure S1. Comparison of temperature coefficient of resistance from recent studies on temperature sensors. The TCRs are expressed in absolute value.

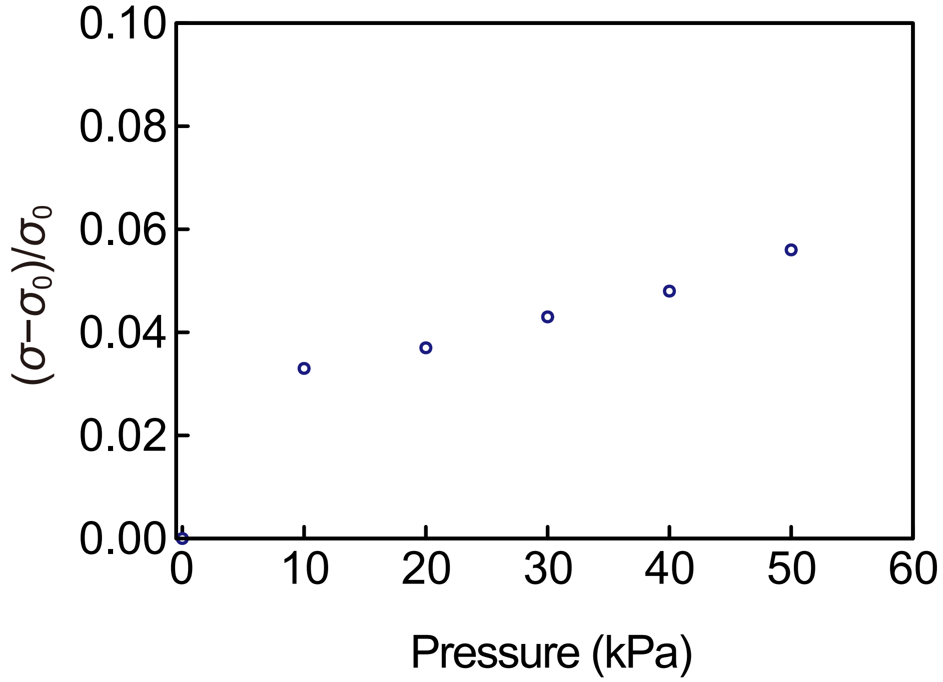

Figure S2. Relative change in conductivity as a function of applied pressure . The pressure-induced variation is negligibly small at 25°C compared to the change observed during temperature sensing. σ and σ_0 represent the ionic conductivity with and without applied pressure, respectively.

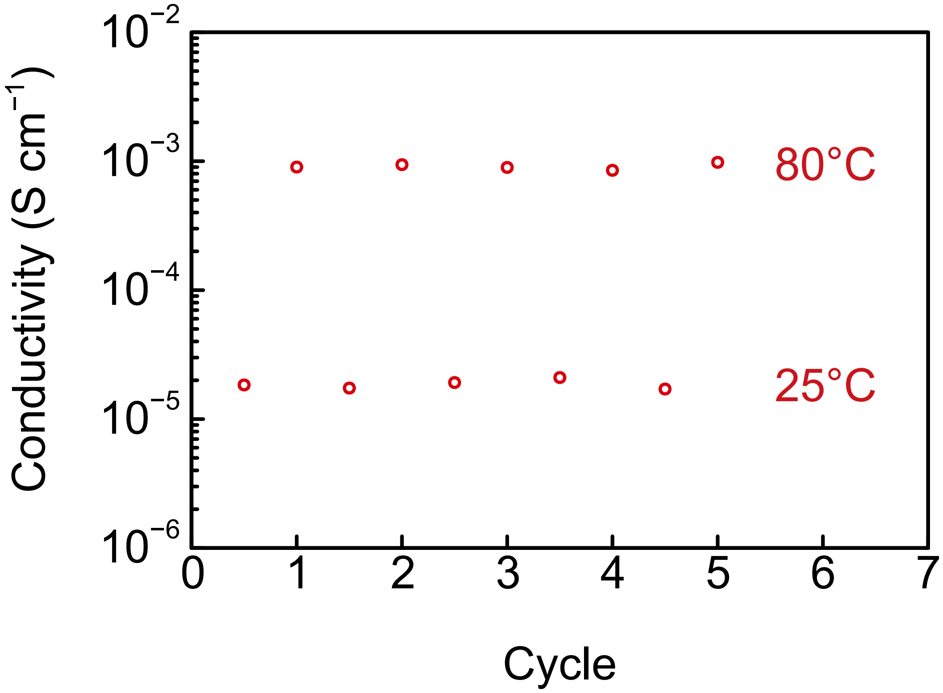

Figure S3. Thermal stability of the temperature sensor. The ionic conductivity was measured while cycling the temperature between 25 °C and 80 °C for five cycles. The conductivity remained stable throughout, indicating no significant degradation of sensor performance.

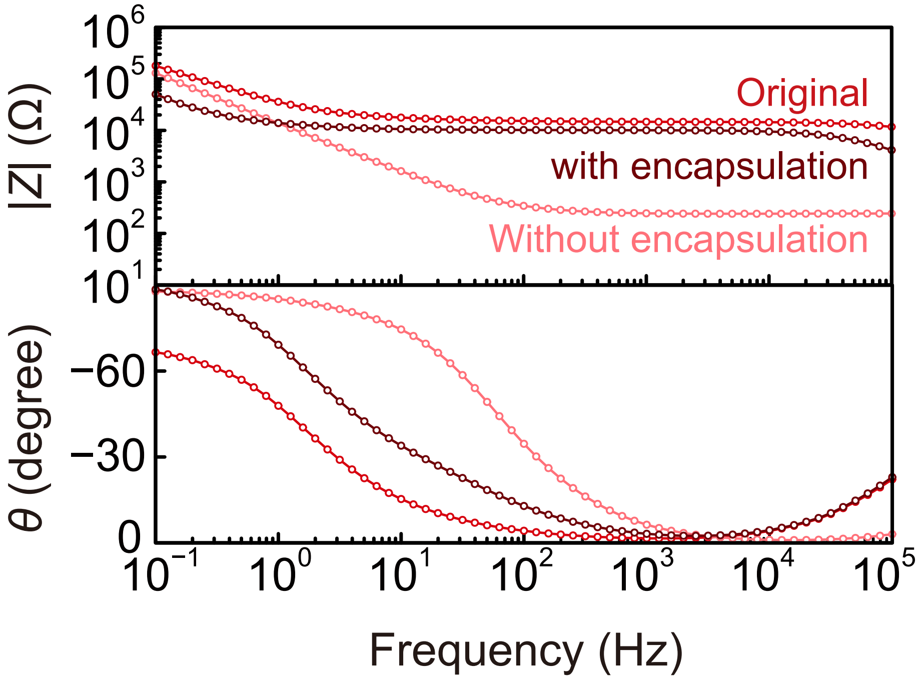

Figure S4. Influence of ambient humidity on temperature sensors with and without an encapsulation layer. The sensor without EPPOMaC encapsulation exhibited noticeable changes in the EIS spectra after 5 days of storage at room temperature (~25 °C), attributed to moisture absorption by the IGNa10. In contrast, the encapsulated sensor showed significantly smaller spectral shifts, indicating that the EPPOMaC layer suppressed moisture uptake and helped preserve the ionic gel's properties.
